# Translation and cross-cultural adaptation of the pediatric cerebral performance category (PCPC) and pediatric overall performance category (POPC) to Brazilian Portuguese

**DOI:** 10.1590/1984-0462/2023/41/2022030

**Published:** 2023-05-29

**Authors:** Talita de Castro Chiarastelli, Cristina dos Santos Cardoso de Sá, Cristiane Sousa Nascimento Baez Garcia, Soraia Libório Cabó, Raquel de Paula Carvalho

**Affiliations:** a Universidade Federal de São Paulo, Santos, SP, Brazil.; b Instituto Federal de Educação, Ciência e Tecnologia do Rio de Janeiro, Rio de Janeiro, RJ, Brazil.; c Fundação José Luiz Egydio Setubal, São Paulo, SP, Brazil.

**Keywords:** Translations, Child, Pediatric intensive care unit, Morbidity, Traduções, Criança, Unidade de terapia intensiva pediátrica, Morbidade

## Abstract

**Objective:**

To translate and culturally adapt the scales Pediatric Cerebral Performance Category (PCPC) and Pediatric Overall Performance Category (POPC) to the Brazilian population.

**Methods:**

Two English language proficient professionals independently translated the original version of the scales into Brazilian Portuguese. After consensus, it was generated a translated version of each scale. These were back translated into English by two native English translators. A new consensus process resulted in an English version of each scale, which were compared with the originals and approved by the author. A committee of experts with clinical and academic experience in intensive care checked the validity of the content and produced the pre-final versions of the scales, which were tested by 25 professionals from a Pediatric Intensive Care Unit. An audit was conducted to verify the consistency of the methodological process.

**Results:**

The pre-final versions were approved by 96% of the Brazilian professionals. No significant changes were made to the content of the instrument; however, it was identified the need of a guide with instructions on how to use the scales.

**Conclusions:**

The process of translation and cross-cultural adaptation of the scales was completed and resulted in PCPC-BR and POPC-BR scales.

## Resumo

**Objetivo:**

Traduzir e adaptar culturalmente as escalas *Pediatric Cerebral Performance Category* e *Pediatric Overall Performance Category* para a população brasileira.

**Métodos:**

Dois tradutores proficientes na língua inglesa traduziram, independentemente, as versões originais das escalas para o português brasileiro. Após consenso, gerou-se uma versão traduzida de cada escala. Estas foram retrotraduzidas para o inglês por dois tradutores nativos da língua inglesa. Um novo consenso resultou em novas versões em inglês de cada escala, que foram confrontadas com as originais e receberam a aprovação da autora. Uma comissão de especialistas com experiência clínica e acadêmica em terapia intensiva verificou a validade de conteúdo e gerou as versões pré-finais das escalas, que foram testadas por 25 profissionais de uma Unidade de Terapia Intensiva Pediátrica. Uma auditoria foi realizada para verificar a consistência do processo metodológico.

**Resultados:**

As versões pré-finais foram aprovadas por 96% dos profissionais brasileiros. Não foram necessárias mudanças importantes no conteúdo do instrumento, entretanto observou-se a necessidade da criação de um guia com instruções sobre a aplicação das escalas.

**Conclusões:**

O processo de tradução e adaptação cultural das escalas foi concluído e resultou nas escalas PCPC-BR e POPC-BR.

## INTRODUCTION

Since its conception, the Pediatric Intensive Care Unit (PICU) has played a paramount role in the health care of critically ill children, with constant improvement through technological advances, increased understanding of disease pathophysiology, and the development of multidisciplinary work.^1^ Children admitted to the PICU have a heterogeneous mix of conditions^1,2^ and may present outcomes, such as the emergence of new physical, psychological, cognitive, and social morbidities^3,4^, due to events occurred during their hospitalization, at the time of hospital discharge, or in the long term.^4,5^ Examples of new morbidities are respiratory dysfunction, pain, decreased sensitivity, low mobility, delayed neuropsychomotor development, paresis, difficulties with personal care and feeding, sleep changes, fatigue, weakness, sleep anxiety, intellectual deficit, attention and/or memory, behavioral changes and decreased academic performance.^5,6^

For many years mortality was the unique isolated parameter for assessing the quality of care. However, the discussion about the association of morbidity measures has been broadened to complement the evaluation of the outcome of patients in PICU.^7^ Historically, three approaches have been widely used to evaluate this outcome: quality of life, multidimensional and adaptive behavior, and global morbidity behavior assessments.^3,7^

Among the instruments used for the global morbidity assessment are the Pediatric Cerebral Performance Category (PCPC) and the Pediatric Overall Performance Category (POPC) scales, that quantify cognitive deficit and general functional morbidity, respectively. The PCPC and POPC were based on the Glasgow Outcome Scale (GOS)^8^ for children. Although GOS consists of only five categories, a category for mild disability was included in POPC and PCPC on the premise that even mild functional impairment in children may be significant based on its duration and impact on neuropsychomotor development.^8^

PCPC and POPC were also based on the impressions of observers who scored child's cognitive deficit and general functional morbidity from one to six. Thus, the scores consider 1 for good, 2 for mild disability, 3 for moderate disability, 4 for severe disability, 5 for vegetative state or coma, and 6 for death. The higher scores represent progressively higher functional impairment. They were developed and validated for use in the hospital environment, presenting a high degree of reliability among evaluators (r=0.88–0.96)^8^, and are sensitive to detect longitudinal changes in the child's functional status during hospitalization. These scales are interrelated once the PCPC score is included in the description of the POPC categories. The administration of the scales is quick and easy, which allows the study of trends through the collection of a large amount of population data.^3^ No specific training is required, but the professional should be familiar with child neuropsychomotor development. The scales have been used in large pediatric studies^8,9,10,11,12,13^ and their scores were related to other morbidity measures such as length of stay in the PICU, total hospitalization expenses, need for post-discharge care, and degree of disease severity.^8,9–13^ In addition, PCPC also showed a positive correlation with psychometric measurements of the Stanford-Binet Intelligence Scale and Bayley Mental Developmental Index, and POPC, with the Bayley Mental Developmental Index and Vineland Adaptive Behavior Scale.^9^

Despite their relevance and easy application, the scales are not available to the Brazilian population in a translated and culturally adapted version. We consider that the translation and cultural adaptation of assessment instruments have many advantages over the creation of new measures^14,15^ and allow, when carefully validated, the comparison of data from studies conducted in different countries, and the exchange of information among researchers.^16^ Therefore, this study aimed to translate and culturally adapt the PCPC and POPC scales to Brazilian Portuguese.

## METHOD

**Figure 1.**
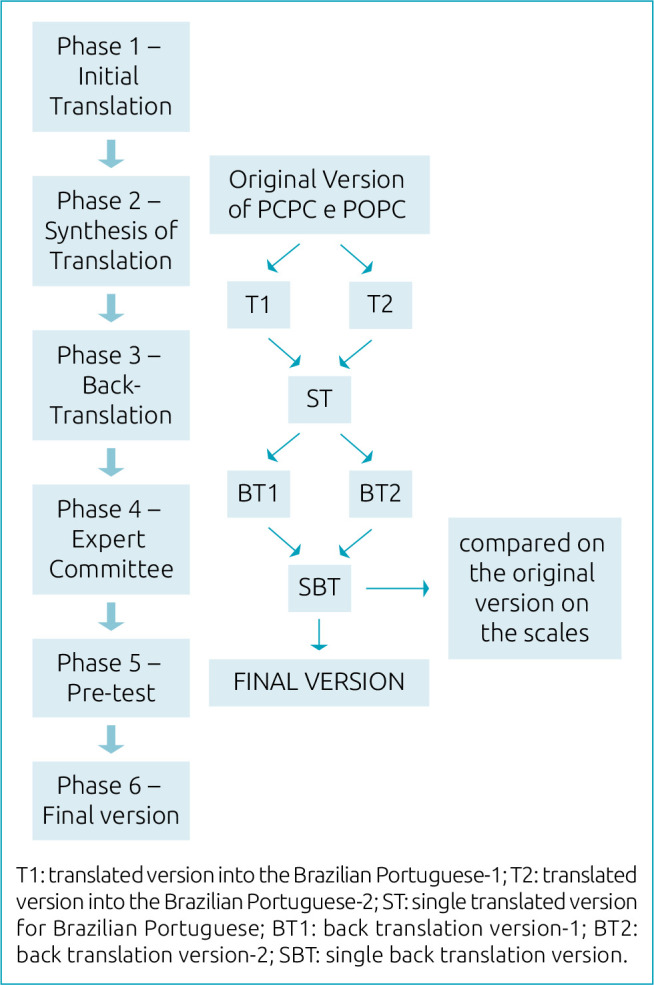
Methodological process flowchart.

The translation and cultural adaptation of the PCPC and POPC scales were carried out with the author's formal authorization and followed the methodological steps recognized internationally, which are:

Phase 1 – Initial translation,
Phase 2 – Synthesis of translations,
Phase 3 – Back translation,
Phase 4 – Expert Committee,
Phase 5 – Pre-test, and
Phase 6 – Final version (Figure 1).^14,15^

The original scales presented in English were translated into Brazilian Portuguese in Phase 1 by two Brazilian translators with different profiles and proficiency in the English language. One professional had expertise in the health area and was aware of the concepts examined in the instruments but not familiar with the scales, and the other had no training in the health area and was unfamiliar or unaware of the scales. This phase resulted in two versions in Brazilian Portuguese, T1 and T2, and a documentary report of this process.

In Phase 2, the versions T1 and T2 were synthesized in a single version through consensus among the researchers of the study and based on the previous documentary report. This phase resulted in a single version for Brazilian Portuguese called single translation (ST). Subsequently, the ST version was back translated into English by two translators who had English as their mother language, also fluent in Brazilian Portuguese, with no training in the health area. That was Phase 3, called back translation (BT), and resulted in two versions BT1 and BT2 and another documentary report. A new discussion was conducted among the researchers, based on the documentary report of Phase 3, and resulted in a single version, called single back translation (SBT), which was forwarded to the author of the scales in table format, comparing with the original version. After the author's approval, Phase 4 was initiated, and the SBT and ST versions were forwarded to a committee of bilingual experts, a nurse and two physiotherapists, with clinical and academic experience in pediatric intensive care, who evaluated semantic, idiomatic, conceptual and experiential equivalence through a questionnaire, according to Beaton et al.^15^ Semantic equivalence refers to grammatical and vocabulary evaluation of each item, preserving the formulation of terms and the equivalence of meanings. Language equivalence evaluates the idiomatic and colloquial expressions that are difficult to translate. Experiential equivalence evaluates whether items express specific cultural experiences using terms consistent with the cultural reality of the population. Conceptual equivalence evaluates words with different concepts in both cultures from original and translated scale.^16^ Equivalences of the items from PCPC e POPC scales were scored as: Totally Adequate (TA), Adequate (A), Inadequate (I), and Totally Inadequate (TI). The experts were allowed to include comments and suggestions in the descriptions about equivalence evaluations.

The considerations of the experts were compiled, and a new consensus emerged among the researchers, who advanced with the pre-final version (PFV) of the scales called PCPC-BR and POPC-BR. In Phase 5, the PFV was tested by 25 physiotherapists in a co-participant center. Each physiotherapist, with at least two years of experience in the area, twice evaluated a distinct child, who stayed a minimum of 48 hours in the PICU. The first evaluation occurred at the time of admission and the second, at the time of the PICU discharge. After the second evaluation, each physiotherapist answered a questionnaire that assessed their degree of satisfaction with the scales. The documentation of the entire process of translation and cultural adaptation was reviewed by the researchers of the study and forwarded to the co-participant center in Phase 6, to ensure that the methodological process was followed and the final version of scales was obtained.

## RESULTS

After obtaining the ST and SBT version, the author and the researchers verified that there was no need to change the content of the scales.

The experts evaluated the semantic equivalence of PCPC, scored on 67 items (74%) as TA, 14 items (16%) as A, 9 items (10%) as I, and no item as TI. Regarding idiomatic equivalence, 39 items (65%) were scored as TA, 4 items (7%) as A, 17 items (28%) as I, and no item was considered TI. On experiential equivalence, 28 items (93%) were scored as TA, 1 (3%) as A, 1 (3%) as I, and no item as TI. Conceptual equivalence scored 27 items (90%) as TA, 1 item (3%) as A, 2 items (7%) as I, and no item as TI. All items considered inadequate were reappropriated.

The changes indicated by the experts in the PCPC scale are presented in Table 1. In the title, the specialist “A” pointed out grammatical difficulty and the need to formulate an equivalent idiomatic expression. The specialist “C” proposed a change in the title but did not point out inadequacies in the translation. In the description of score 6, the specialist “A” suggested replacing “brain death” with “encephalic death”, but the group decided to maintain the brain term, as defined in the original scale creation.

**Table 1.** Changes made in de single translation to create the pre-final version of the Pediatric Cerebral Performance Category based on expert suggestions.

Item	ST	PFV
Título	Escala de categoria de desempenho cerebral pediátrico	Escala de categorização de desempenho cerebral pediátrica – (PCPC-BR)
Deficiência leve	A série talvez não seja apropriada para a idade	O ano talvez não seja adequado a idade
Deficiência moderada	Função cerebral suficiente para atividades independentes da vida diária apropriadas à idade	Função cerebral suficiente para realizar de forma independente atividades de vida diária apropriadas à idade
Coma ou estado vegetativo	“Mesmo se aparentar despertar”	“Mesmo se aparentar estar acordado”
	“Nenhuma evidência de função do córtex (não ativação por estímulos verbais)”	“Nenhuma evidência de função cortical (sem resposta a estímulos verbais)”

ST: single translation; PFV: pre-final version; PCPC-BR: Pediatric Cerebral Performance Category (Brazilian Portuguese version).

In the description of the categories, semantic and idiomatic difficulties were observed in the translation of score 1 – “normal” by the specialist “A”, score 2 – “mild deficiency” by specialist “A” and “B”, and score 3 – “moderate deficiency” by specialist “A”. The specialist “C” pointed out conceptual inadequacy of score 3. Specialist “B” pointed out the need to correct the description of score 4 – “severe deficiency”, due to idiomatic, conceptual and experience divergences. For score 5 – “coma state or vegetative state”, specialist “A” indicated idiomatic inadequacy while specialist “C” indicated semantic and idiomatic inadequacies. Idiomatic and semantic inadequacies in score 6 – “brain death” were reported by specialist “A”.

The semantic equivalence of POPC was scored by the experts in 48 items (76%) as TA, 14 items (22%) as A, 1 item (2%) as I, and no item as TI. Regarding idiomatic equivalence, 28 items (67%) were scored as TA, 8 items (19%) as A, 6 items (14%) as I, and no item were considered TI. On experiential equivalence, 19 items (90%) were scored as TA, no item as A, 2 items (10%) as I, and no item as TI. Conceptual equivalence scored 19 items (90%) as TA, 2 items (10%) as I, and no item as A or TI.

The changes made in the POPC scale are presented in Table 2. Specialist “A” indicated idiomatic inadequacy in the title and description of scores, suggesting a change in the term “brain death” to “encephalic death”. In score 2 – “mild general deficiency”, idiomatic inadequacy was pointed out by specialist “A” and semantics by specialist “B”. Specialist “A” suggested change to score 3 – “moderate general deficiency” and score 4 – “severe general deficiency”. In turn, the specialist “B”, considered idiomatic, experiential and conceptual inadequacies in the description of score 3.

**Table 2.** Changes made in de single translation to create the pre-final version of the Pediatric Overall Performance Category based on expert suggestions.

ITEM	ST	PFV
Título	Escala de categoria de desempenho geral pediátrica	Escala de categorização de desempenho geral pediátrica (POPC-BR)
Deficiência leve	“Consciente e capaz de independência de forma funcional”	“Consciente e independente de forma funcional”
Deficiência moderada	“Devido à disfunção dos sistemas não-cerebrais isoladamente ou com disfunção do sistema cerebral”	“Devido à disfunção isolada de sistemas não-cerebrais ou com disfunção do sistema cerebral”
	“É desabilitado para desempenho competitivo na escola”	“Mas não possui habilidade para o desempenho competitivo na escola.”
Deficiência severa	“Por disfunção dos sistemas não-cerebrais isoladamente”	“Devido à disfunção isolada de sistemas não-cerebrais”

ST: single translation; PFV: pre-final version; POPC-BR: Pediatric Overall Performance Category (Brazilian Portuguese version).

Twenty-five patients participated in the PFV test, 25 physiotherapists with an average of 7.8±5.5 years of physical therapy training and 5.2±5.0 years of experience in PICU. They all had specialization in different areas reported in child health (Pediatric Intensive Care, Pediatric Oncology, Cardiovascular Physiotherapy and Respiratory Physiotherapy). Regarding previous knowledge of the scales, 88% did not know, 12% knew, and 4% had already used them. Only 1 profissional (4%) had previous experience with translation and cultural adaptation of scales.

Regarding PCPC-BR and POPC-BR, respectively, 96% and 96% of the professionals believed that the content was adequate for the context of the Brazilian child, 84% and 88% that the content was adequate for the Brazilian child in the established age group, 96% and 96% that the translation was performed in a clear and cohesive way and that the cultural adaptation was performed appropriately, and 92% and 84% judged that no item of the scales required alteration.

When the professionals were asked about the need for changes, they cited mainly the age group established, both for PCPC-BR and POPC-BR. Specifically for POPC-BR, they observed the need to describe the severity of cognitive deficit of the children evaluated in scores 5 and 6.

After the end of the study, the documentation of the entire process of translation and culture adaptation was approved by the co-participant center. Tables 3 and 4 include the PCPC-BR and POPC-BR scales. The guidelines for application are in Table 5.

**Table 3.** Brazilian version of Pediatric Cerebral Performance Category scale.

Escala de categorização de desempenho cerebral pediátrica – (PCPC-BR)		
Pontuação	Categoria	Descrição
1	Normal	Normal; no nível apropriado da idade; criança em idade escolar que frequenta a escola regular.
2	Deficiência leve	Consciente, alerta e capaz de interagir no nível apropriado para a idade; criança em idade escolar frequentando a escola regular, mas o ano talvez não seja adequado a idade; possibilidade de déficit neurológico leve.
3	Deficiência moderada	Consciente; função cerebral suficiente para realizar de forma independente atividades de vida diária apropriadas à idade; criança em idade escolar que frequenta sala de aula de educação especial e/ou déficit de aprendizagem presente.
4	Deficiência grave	Consciente; dependente de outras pessoas para suporte diário por causa da função cerebral prejudicada.
5	Coma ou estado vegetativo	Qualquer grau de coma sem a presença de todos os critérios de morte cerebral; inconsciência, mesmo se aparentar estar acordado, sem interação com o ambiente; sem responsividade cerebral e nenhuma evidência de função cortical (sem resposta a estímulos verbais); possibilidade de alguma resposta *reflexa*, abertura ocular espontânea e ciclos de sono-vigília.
6	Morte cerebral	Apneia, arreflexia e/ou silêncio eletroencefalográfico.

PCPC-BR: Pediatric Cerebral Performance Category (Brazilian Portuguese version).

**Table 4.** Brazilian version of Pediatric Overall Performance Category scale.

Escala de categorização de desempenho geral pediátrica (POPC-BR)		
Pontuação	Categoria	Descrição
1	Bom desempenho geral	PCPC 1: Saudável, alerta e capaz de realizar atividades normais de vida diária
2	Deficiência geral leve	PCPC 2: Possibilidade de pequeno problema físico ainda compatível com a vida normal; consciente e independente de forma funcional
3	Deficiência geral moderada	PCPC 3: Possibilidade de deficiência moderada devido à disfunção isolada de sistemas não-cerebrais ou com disfunção do sistema cerebral; consciente e realiza atividade de vida diária de forma independente, mas não possui habilidade para o desempenho competitivo na escola
4	Deficiência geral grave	PCPC 4: Possibilidade de deficiência grave devido à disfunção isolada de sistemas não-cerebrais ou com disfunção do sistema cerebral; consciente, mas dependente dos outros para atividades da vida diária
5	Coma ou estado vegetativo	PCPC: 5
6	Morte cerebral	PCPC: 6

POPC-BR: Pediatric Overall Performance Category (Brazilian Portuguese version); PCPC: Pediatric Cerebral Performance Category.

**Table 5.** Instruction for the application of the Brazilian version of the Pediatric Cerebral Performance Category and Pediatric Overall Performance Category scales.

Instruções para aplicação das versões brasileiras escalas POPC e PCPC
A Escala de Categorização de Desempenho Cerebral Pediátrica – (PCPC-BR) e a Escala de Categorização de Desempenho Geral Pediátrica (POPC-BR) são versões traduzidas para o Português brasileiro das escalas *Pediatric Cerebral Performance Category* (PCPC) e *Pediatric Overall Performance Category* (POPC), desenvolvidas pela médica Debra H. Fiser. A PCPC-BR e POPC-BR são originalmente desenvolvidas com o objetivo de quantificar, em curto prazo, o prejuízo cognitivo (PCPC-BR) e a morbidade funcional global (POPC-BR). Cada categoria das escalas é acompanhada de descrições operacionais direcionadas para a idade da criança no momento da avaliação. A escala POPC-BR depende da escala PCPC-BR, pois a pontuação da escala PCPC-BR está incluída na descrição da categoria da escala POPC. Durante a aplicação do instrumento, o avaliador deverá pontuar o pior nível de desempenho observado na criança, de acordo com as descrições. Os déficits são pontuados na escala PCPC-BR, caso resultem de alterações neurológias. Na POPC-BR os déficits são pontuados se resultarem de alterações neurológicas (*status* PCPC-BR), ou outras doenças e condições (como por exemplo asma ou amputações).
População: Crianças admitidas em Unidade de Terapia Intensiva Pediátrica (UTIP). A idade vai depender dos critérios estabelecidos pela UTIP. Os estudos de Fiser apontam para uma faixa etária de 0 a 21 anos.
Avaliador: Deverá ser um profissional da equipe multidisciplinar (médico, enfermeiro, fisioterapeuta). Não há necessidade de um treinamento específico para se aplicar as escalas, porém o profissional deve ter conhecimento prévio no desenvolvimento infantil.
Avaliação: As escalas podem ser aplicadas por meio de entrevista com pais, responsáveis, médico ou cuidador da criança. A avaliação de prontuários é uma alternativa válida. O pior nível de desempenho para qualquer critério da descrição é utilizado para categorizar o *status* da PCPC-BR e POPC-BR. Recomenda-se duas aplicações, sendo a primeira no momento da admissão e a segunda no momento alta da UTIP. A partir das duas avaliações é obtido o delta escore, calculado por meio da subtração do resultado da segunda avaliação pelo resultado da primeira.
Pontuação: Ambas as escalas apresentam seis pontuações, sendo que as maiores pontuações representam um prejuízo funcional progressivamente maior. A PCPC - BR possui 6 categorias sendo 1- Normal; 2- Deficiência Leve; 3- Deficiência modera; 4- Deficiência grave; 5-Coma ou estado Vegetativo; 6-Morte Cerebral. A POPC- BR irá predizer o desempenho cerebral como 1- Bom desempenho global; 2- Deficiência global leve, 3-Deficiência global moderada; 4-Deficiência global grave; 5-Coma ou estado vegetativo; 6- Morte cerebral. Escore delta (Δ) é calculado a partir da diferença de pontuação entre a segunda e a primeira avaliação da criança e demonstra o reflexo direto da mudança na capacidade funcional da criança após um episódio de injúria e internação em UTIP. SEGUNDA AVALIAÇÃO−PRIMEIRA AVALIAÇÃO=SCORE DELTA (Δ) O escore Delta zero demonstra que não houve mudança no status de capacidade funcional após internação. $$\begin{array}{l} \text{Exemplo:}\\ \text{Primeira avalia}çã\text{o a pontua}çã\text{o PCPC BR e POPC- BR = 3}\\ \text{Na segunda avalia}çã\text{o a pontua}çã\text{o PCPC BR e POPC- BR = 3}\\ \Delta =3-3,\text{ou seja,}\Delta =0-\text{N}ã\text{o observada mudan}ç\text{a no status funcional da crian}ç\text{a} \end{array}$$ Um valor positivo demonstra aumento de degradação da capacidade funcional. Um valor negativo indica melhora em relação ao estado prévio a admissão. $$\begin{array}{l} \text{Exemplo:}\\ \text{Primeira avalia}çã\text{o a pontua}çã\text{o PCPC BR e POPC- BR = 2}\\ \text{Na segunda avalia}çã\text{o a pontua}çã\text{o PCPC BR e POPC- BR = 3}\\ \Delta =3-2,\text{ou seja,}\Delta =1-\text{A crian}ç\text{a apresenta uma piora em rela}çã\text{o a avalia}çã\text{o inicial} \end{array}$$ Em caso de readmissão na UTIP antes da alta hospitalar, ou realização de acompanhamento pós alta hospitalar, a pontuação da última avaliação serve como base para o cálculo do *escore delt*a.

## DISCUSSION

This study translated and adapted for the Brazilian Portuguese the PCPC and POPC scales – that aim to describe in the short term the outcome of pediatric intensive care, quantifying general functional morbidity and cognitive deficit –, resulting in the PCPC-BR and POPC-BR versions. We chose to base the study design on the methodology^15^ composed of the initial translation procedures, translation synthesis, back translation, expert committee, pre-test and final version, since we understand that translation and cross-cultural adaptation is a delicate process. In addition, to achieve equivalence between the original instrument and the translated and adapted versions, it is necessary to follow a rigorous process.

The phase that refers to the translation from English to Brazilian Portuguese did not demonstrate divergences or inconsistencies with important impact on the *constructor*. Only a few disagreements were observed between translators regarding verbal tense, nominal agreement, use of synonymous words, such as “score”, “appropriate”, “performance”, and technical terms, such as “sleep-wake cycle” and “areflexia”.

These disagreements were already expected,^15^ since the translators, despite being bilingual, had different backgrounds. The first was aware of the concepts explored in the instruments and had training in the health area, which gives a clinical and more equivalent perspective to the translated content. The second translator had no training in the health area, nor knowledge about the content.

During the execution of the synthesis of the translations, we analyzed the first phase report of both translators and performed the fusion of the translations, according to the need on items that, although not incorrect, could hinder the understanding of the information. One might argued that at this time it would be necessary for professional translators to intervene due to their language skills. However, this choice did not make the translation process impossible because at that time familiarity with the area was more recommended than with the grammatical optimization of the target culture, and this option was also supported in the literature.^14^

We found some controversies in the literature regarding the need for the back-translation phase. Epstein et al.^16^ indicated that back translation could have limited use, particularly if the adaptation team has proficiency in the languages of origin and target culture. However, in our study, we concluded that this phase allowed the involvement of the author of the scales, besides demonstrating that there were no discrepancies that prevented the continuity of the process.

The experts committee phase proved to be important due to contributions and criticism, as well as the aid to reach consensus at the time of consolidation of previous versions into a single one.^17^ The role of the expert committee is to consolidate all versions of the questionnaire and assist in the development of the preliminary version of the scale translation.^15^ Changes were necessary in the ST version to obtain the PFV, because some items, although not improperly translated, were difficult to understand and interpret, as reported before.^18^

The instructions for applying PCPC and POPC are described by the author in the publications on the scales.^8-10^ Considering the need for access to these guidelines by Brazilian professionals, we gathered the information related to the application of the scales in a document called “Instruction for the application of the PCPC-BR and POPC-BR scales” (Table 5). Although this document is not part of the translation process, we understand that it was a necessary cultural adaptation, since the information is available in different articles published in English, which could hinder the use of the Brazilian version of the scales. The instructions were also submitted to be analyzed by the committee of experts, and the application, by the physiotherapists.

Regarding the inadequacies pointed out by the specialists, these were qualitatively evaluated and resulted in changes to obtain the final version (FV). The changes were made in the title and description of the scores. For the title, we chose to maintain the original acronym of the scale with the identification “BR” in order not to lose the characteristic of the original instrument, to facilitate the identification and understanding of the scales, and optimize the search in the databases for studies with application of the scales.

The application phase of the translated version of the scales also proved to be important in the process of cultural adaptation, as it provided a better understanding about the perspective of professionals with training and action focused on the Brazilian reality. The level of satisfaction in relation to the translated and adapted version of the scales was high. Nevertheless, suggestions were made to increase clarity of the information on the established age group with the term “school phase”, and in the description of the scores “normal”, “mild deficiency” and “moderate deficiency” of the PCPC-BR scale. In addition, it was proposed to include the PCPC scale score in the operational definitions of the POPC scale.

Regarding the established age group, the physiotherapists related the term “school phase” as mandatory when referring to a child old enough to attend school, which, according to them, would prevent the application in children who are not old enough yet. However, the scales use the term “school phase” as a reference for the chronological age of the child at the time of evaluation, inducing the professional to identify the stage of development in which the child is, regardless of whether or not he/she attends school. Global morbidity assessment scales, such as PCPC and POPC, have the advantage of being easy and fast to administer, which allows their use in large population studies to assess trends. However, these scales are subjective and require from the evaluators the ability to identify appropriate developmental milestones for the age group.^3^

Another concern of the professionals was whether there would be indication of the POPC scale for patients with important cognitive deficit. The answer is yes because identifying, even subjectively, the patient's cognitive level allows a better understanding of the clinical picture, moreover, minimizes the risk of bias in studies when assigning previous disabilities as resulting from some intervention or disease. Aliev et al. observed that 46% of the patients evaluated had, at the time of hospitalization, some degree of cognitive disability and that, at the time of discharge, this number was 60%.^12^ There are specific scales for assessing cognitive impairment and adaptive behavior, but many require specific training and longer time for application. The POPC-BR scale emerges as an alternative for the overall assessment of cognitive impairment when the use of specific measures is not the first choice.

The results of this study demonstrated good acceptance of the PCPC-BR and POPC-BR scales by Brazilian professionals. However, some limitations should be considered, such as the failure to evaluate psychometric properties in the Brazilian version of scales, evaluation in a single hospital and verification by only one area of the multidisciplinary team. We suggest future studies on the psychometric properties evaluation of PCPC-BR and POPC-BR, as a guarantee for their application in clinic and in several studies.

As a conclusion, the translation and cultural adaptation phases of the scales were completed, resulting in the PCPC-BR and POPC-BR versions. Further studies are necessary to evaluate the validity and reliability to ensure their precision use in the Brazilian population.

## Data Availability

The database that originated the article is available with the corresponding author.
